# Decoding Dyspnea: Unveiling Malignancy Disguised as Asthma

**DOI:** 10.7759/cureus.89410

**Published:** 2025-08-05

**Authors:** Vishaka R Hatcher, Veronica C Alix, Sandy K Yip, Karla Adams

**Affiliations:** 1 Allergy and Immunology, Wilford Hall Medical Center, San Antonio, USA; 2 Allergy and Immunology, Southern Nevada Allergy, Henderson, USA

**Keywords:** asthma, cancer, chronic cough, cough, diagnostics, dyspnea, lower airway disease, spirometry, upper airway disease

## Abstract

We present two patients who presented with symptoms that overlap with asthma, but upon further diagnostic evaluation, were revealed to have underlying malignancy. These cases highlight the importance of objective evidence-based evaluation in unveiling diagnoses previously mislabeled as asthma. The first patient was a 51-year-old with one year of cough and waning albuterol responsiveness, with worsening orthopnea and exertional dyspnea. Spirometry demonstrated a mixed obstructive/restrictive pattern without a bronchodilator response. Chest radiograph, which was not previously obtained, revealed left-sided nodules and right-sided consolidation, with computed tomography and specialist evaluation leading to the diagnosis of aggressive lung cancer. The second patient was a 24-year-old with one month of new dyspnea with marginal response to albuterol, improvement on oral steroids, and dysphagia. Spirometry revealed a fixed obstructive pattern without a bronchodilator response. Chest radiograph was within normal limits. Neck computed tomography revealed a large neoplasm involving the esophagus, resulting in tracheal displacement and compression. In addition to detailed history, exam, and spirometry, diagnostic evaluation of asthma should include consideration of other possible causes for obstruction. The first patient highlights how presumptive diagnoses can endure and delay accurate diagnosis and treatment, thereby increasing morbidity. The second case highlights that although asthma is a more likely diagnosis than malignancy in a healthy young adult, systematic evaluation may reveal critical previously missed findings. These cases remind clinicians that uncommon and sinister diseases can masquerade as diseases that we commonly manage, and illuminate the importance of methodical evaluation with objective evidence to diagnose and treat asthma.

## Introduction

Asthma is a complex disorder with a diverse spectrum of symptoms that share key overarching characteristics [1-4]. The definition of asthma has long been debated, and thought to not be one disease, but instead a heterogeneous constellation of phenotypes and endotypes that share common features of bronchial hyperresponsiveness, chronic airway inflammation, and variable obstructive ventilatory impairment [1,3,4]. As symptoms alone do not accurately predict pulmonary function, there is evidence of both overdiagnosis and underdiagnosis of asthma in the adult population [4]. Diagnosis relies on the clinical history and objective evidence of bronchodilator response.

We present two patients who presented with symptoms that overlap with asthma, but upon further diagnostic evaluation, were revealed to have underlying malignancy. These cases highlight the importance of objective evaluation in combating anchoring bias and unveiling diagnoses previously mislabeled as asthma. 

The abstract of this article was previously presented as a poster at the American College of Allergy, Asthma & Immunology (ACAAI) annual meeting in October 2024 in Boston, Massachusetts, United States.

## Case presentation

Case 1

A 51-year-old female patient presented with chronic perennial rhinitis and one year of cough in the absence of any personal history of tobacco use or exposure to secondhand smoke. She was initially prescribed albuterol by her primary care manager due to a presumptive diagnosis of asthma, in the context of a history of allergic rhinitis symptoms and no social or occupational exposures to explain cough. Sporadic as-needed use of albuterol seemed to initially improve symptoms, but the patient began needing albuterol at least daily for her cough in the past three months, with waning albuterol responsiveness. The patient denied any audible wheezing or mucus production but described an occasional sensation of pressure in her chest with the cough. She had progressive exertional dyspnea and reported her cough was exacerbated when lying on her right side. She denied weight loss, night sweats, and a history of overseas travel before symptom onset. Intramuscular methylprednisolone did not improve symptoms. No chest radiographs had been obtained in the past decade despite her chronic and recently deteriorating symptoms. She was referred to an allergist due to persistent and worsening symptoms, with a presumed diagnosis of asthma in the absence of any objective evaluation.

**Figure 1:**
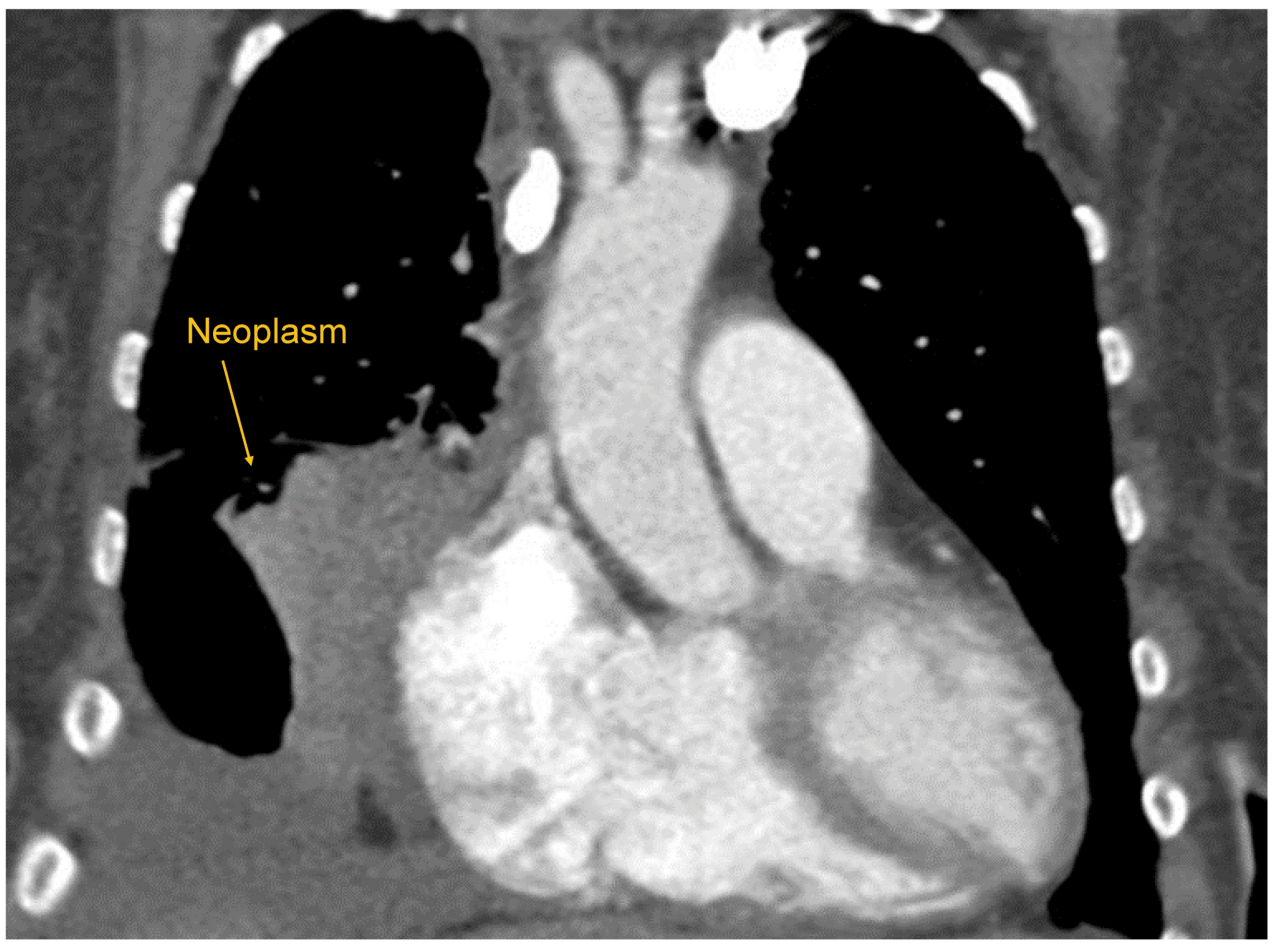
Coronal view of chest CT of a 51-year-old with dyspnea and cough, showing aggressive right-sided lung neoplasm (Case 1)

Her physical exam revealed no abnormalities. Spirometry (Table 1) demonstrated forced expiratory volume in one second (FEV1) and forced vital capacity (FVC) below the lower limit of normal, a normal FEV1/FVC ratio, and no significant bronchodilator response, necessitating lung plethysmography to further delineate possible obstructive or restrictive impairment. Due to possible obstructive ventilatory abnormality, the allergist initiated a four-week trial of budesonide/formoterol inhaler therapy while awaiting complete pulmonary function testing with plethysmography in the pulmonology clinic to elucidate the etiology of spirometry findings and waning albuterol responsiveness of dyspnea and cough. In addition to spirometry, the allergist obtained a chest radiograph, which revealed left-sided nodules and right-sided consolidation. Dedicated chest computed tomography (CT) was ordered urgently, and pulmonology evaluation was expedited to minimize further delays in her care. CT of her chest (Figure 1) and specialist evaluation ultimately led to the diagnosis of stage IV non-small cell lung cancer with aggressive growth and diffuse metastases, including to the brain.

**Table 1:** Patient characteristics and evaluation FEV1, forced expiratory volume in 1 second; FVC, forced vital capacity; LLN, lower limit of normal; CT, computed tomography

Characteristics	Patient 1	Patient 2
Age	51 years	24 years
History	1 year of cough, dyspnea	1 month of dyspnea, dysphagia
Spirometry	FEV1/FVC ratio normal, FEV1 and FVC below LLN, inspiratory curve blunting on flow/volume loop	FEV1/FVC ratio below LLN, FEV1 and FVC below LLN, inspiratory and expiratory blunting on flow/volume loop
Bronchodilator Response	No	No
Chest Radiography	Left-sided nodules and right-sided consolidation	Within normal limits
Dedicated Advanced Imaging	Chest CT: Aggressive neoplasm of the lung, space-occupying consolidation in right mediastinum	Neck CT: Large neoplasm involving the esophagus, displacing and compressing trachea to 3mm diameter

Case 2

**Figure 2:**
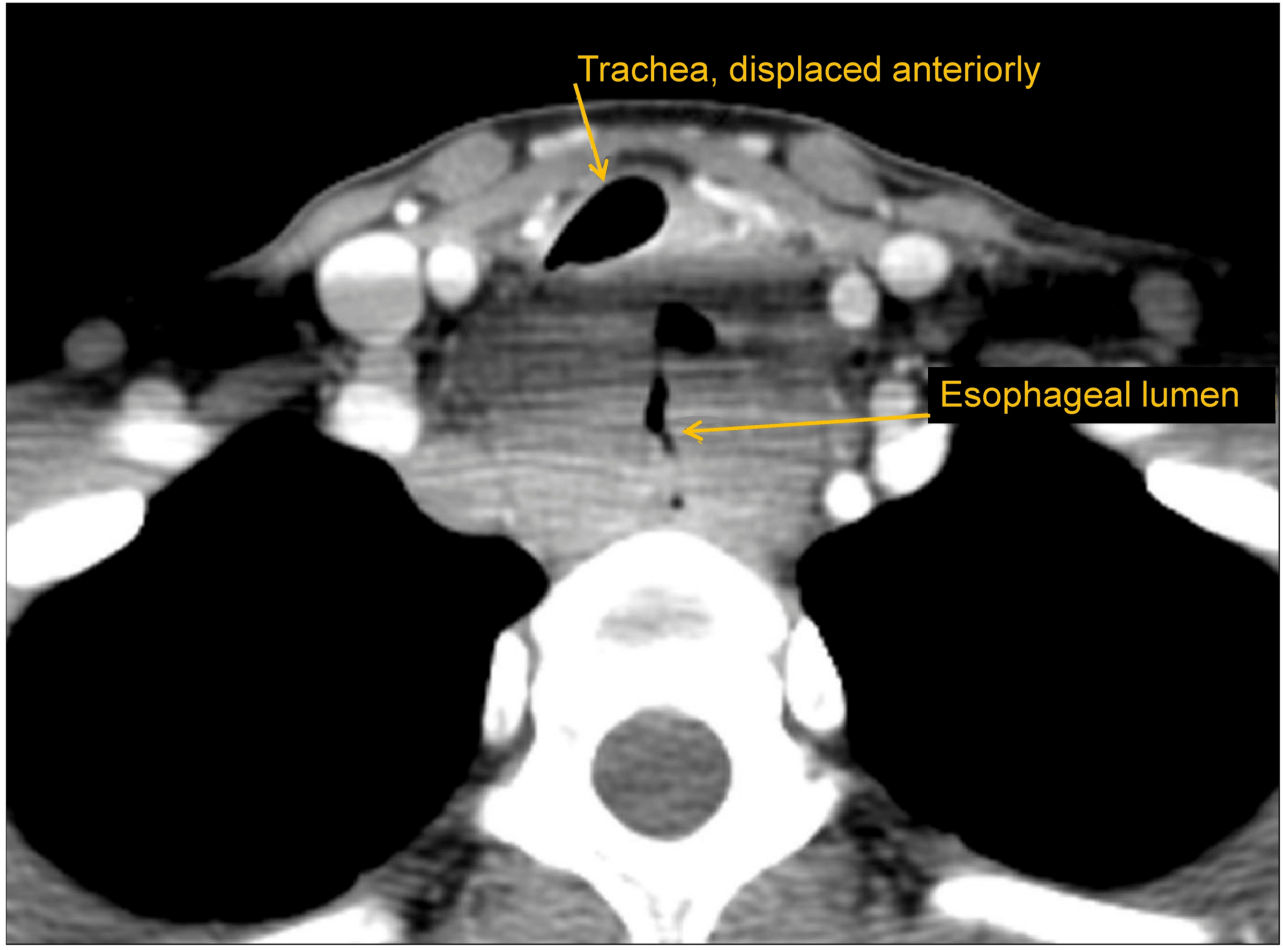
Axial view of neck CT of a 24-year-old with dyspnea and dysphagia, showing large neoplasm involving the esophagus, resulting in tracheal displacement and compression (Case 2)

The second patient was a 24-year-old previously healthy woman with a family history of asthma and one month of new onset dyspnea without cough. Her dyspnea was marginally responsive to albuterol, though improved on oral steroids. The symptoms, however, recurred within 24 hours of oral steroid discontinuation. The patient also complained of globus sensation. She was presumptively diagnosed with asthma based on her family history and lack of complicated past medical history, began on a budesonide/formoterol inhaler, and was advised a trial of an over-the-counter proton pump inhibitor to alleviate possible gastroesophageal reflux contributing to throat discomfort. She subsequently developed new symptoms of snoring and dysphagia, in the absence of obesity or dietary changes. She had normal chest imaging (Table 1) and was referred for allergist evaluation due to continued concern for asthma and possible allergic contribution. Her physical exam was unremarkable. Spirometry performed by the allergist was consistent with a fixed obstructive ventilatory impairment with FEV1 at 57% of predicted. Dedicated neck CT, which was urgently obtained due to concerning spirometry and clinical history, revealed a large neoplasm involving the esophagus, resulting in tracheal displacement and compression (Figure 2). She was immediately referred to otorhinolaryngology and oncology for urgent evaluation, where a diagnosis of non-Hodgkin lymphoma was made and treatment was initiated.

## Discussion

Published guidelines emphasize that, in addition to detailed history, exam, and spirometry, diagnostic evaluation of asthma should include consideration of other possible causes for obstruction [1-3]. The National Asthma Education and Prevention Program’s Expert Panel Report cautions clinicians that presentations of asthma can be heterogeneous and symptoms can vary between patients [3]. Objective assessment of airway obstruction and possible bronchodilator response that would be expected with asthma is best measured through spirometry [1-4]. If spirometry with acceptable efforts does not reveal a reduced FEV1/FVC ratio or a significant bronchodilator response, diagnoses other than asthma should be considered with the complete clinical picture in mind. Although comorbid conditions or unrelated disease processes may not be readily apparent, chest radiography is routinely indicated early in the evaluation process to elucidate any clinical features uncharacteristic of asthma.

The first case highlights how presumptive diagnoses can endure and delay accurate diagnosis and treatment, thereby increasing morbidity and risk of mortality. In both the primary care setting and in specialty clinics, it is important to consider alternative diagnoses, even when a working diagnosis is in place, to avoid an anchoring bias. Anchoring bias occurs when data is prioritized to support an initial impression or diagnosis [5]. Although presentations of dyspnea and chronic cough may present to the specialty care clinic, or even a primary care clinic following an emergency department or urgent care center visit, with presumed diagnosis of asthma, other diagnoses on the differential including systemic disease, malignancy, and anatomic abnormalities of the upper and lower airways should be objectively ruled out prior to initiation of empiric asthma management, especially as untimely use of corticosteroids in a patient with malignancy or infection may not only delay diagnosis but also worsen morbidity associated with disease progression [5-7].

Asthma prevalence has been estimated to affect more than 300 million people worldwide [4]. In developed nations such as the United States, adult asthma prevalence is estimated to be approximately 11% [4]. An estimated 19.3 million new cancer cases occurred worldwide in 2020, and 90% of those were solid tumors [8]. From 2017 to 2021, the incidence of cancer in the United States was 0.46% [9]. Being more common than malignancy, asthma is frequently considered in the differential diagnoses for dyspnea and cough. However, due to the serious morbidity and mortality associated with diagnoses of malignancy, it is of utmost importance to evaluate for and objectively eliminate malignancy as a possible diagnosis prior to empiric treatment of presenting symptoms alone.

The second case in this report demonstrates that although asthma is a more likely diagnosis than malignancy in a healthy young adult, systematic evaluation may reveal critical previously missed findings. Dysphagia and globus sensation are both uncharacteristic of asthma, and fixed obstruction on spirometry should trigger targeted imaging based on the suggested pathologic source (upper airway in this patient, in the absence of chest radiograph abnormalities) and specialist evaluation to arrive at an accurate diagnosis and minimize delays in treatment. Multiple factors may have led to the initial misdiagnosis of asthma in both cases, such as the increased frequency of asthma in females after puberty, which increases the pre-test probability of asthma in such cases [4]. Acknowledging and overcoming an anchoring bias to a diagnosis, especially when a complete evaluation has not been completed, remains critical.

## Conclusions

These cases remind clinicians that uncommon and sinister diseases can masquerade as common diseases and illuminate the importance of consistent and methodical evaluation with objective evidence to accurately diagnose and treat asthma. Prompt, methodical, evidence-based evaluation of all patients presenting with symptoms resembling asthma can support the diagnosis or unveil possible other underlying pathology with minimal delays in diagnosis and treatment. Early use of spirometry and chest radiography in the evaluation of patients with dyspnea and/or cough is critical to accurate and timely diagnosis and treatment. Similarly to symptoms that are not characteristic of asthma, spirometry without evidence of obstruction or bronchodilator response should trigger consideration of diagnoses other than asthma. Although asthma is a prevalent condition managed closely by allergists, pulmonologists, and primary care providers, quelling anchoring biases with astute clinical evaluation can not only prevent misdiagnoses, but also reduce morbidity and mortality associated with delayed diagnosis of serious conditions such as malignancy.
